# Temporal trends in hospitalizations and 30-day mortality in older patients during the COVID pandemic from March 2020 to July 2021

**DOI:** 10.1371/journal.pone.0291237

**Published:** 2023-09-14

**Authors:** Sara Garcia-Ptacek, Hong Xu, Martin Annetorp, Viktoria Bäck Jerlardtz, Tommy Cederholm, Malin Engström, Miia Kivipelto, Lars Göran Lundberg, Carina Metzner, Maria Olsson, Josefina Skogö Nyvang, Carina Sühl Öberg, Elisabet Åkesson, Dorota Religa, Maria Eriksdotter

**Affiliations:** 1 Department of Neurobiology, Division of Clinical Geriatrics, Karolinska Institutet, Care Sciences and Society, Stockholm, Sweden; 2 Theme Inflammation and Aging, Karolinska University Hospital, Stockholm, Sweden; 3 Department of Geriatric Medicine, Jakobsbergsgeriatriken, Stockholm, Sweden; 4 Department of Geriatric Medicine, Sabbatsbergsgeriatriken, Stockholm, Sweden; 5 Department of Geriatric Medicine, Dalengeriatriken Aleris Närsjukvård AB, Stockholm, Sweden; 6 Department of Geriatric Medicine, Capio Geriatrik Löwet, Stockholm, Sweden; 7 Department of Geriatric Medicine, Capio Geriatrik Sollentuna, Stockholm, Sweden; 8 Department of Geriatric Medicine, Capio Geriatrik Nacka AB, Nacka, Sweden; 9 Department of Geriatric Medicine, Handengeriatriken, Aleris Närsjukvård AB, Stockholm, Sweden; 10 Department of Neurobiology, Division of Neurogeriatrics, Karolinska Institutet, Care Sciences and Society, Stockholm, Sweden; 11 R&D Unit, Stockholms Sjukhem, Stockholm, Sweden; University of Modena and Reggio Emilia: Universita degli Studi di Modena e Reggio Emilia, ITALY

## Abstract

**Background:**

A reduction in mortality risk of COVID-19 throughout the first wave of the pandemic has been reported, but less is known about later waves. This study aimed to describe changes in hospitalizations and mortality of patients receiving inpatient geriatric care for COVID-19 or other causes during the pandemic.

**Methods:**

Patients 70 years and older hospitalized in geriatric hospitals in Stockholm for COVID-19 or other causes between March 2020-July 2021 were included. Data on the incidence of COVID-positive cases and 30-day mortality of the total ≥ 70-year-old population, in relation to weekly hospitalizations and mortality after hospital admissions were analyzed.

Findings

The total number of hospitalizations was 5,320 for COVID-19 and 32,243 for non-COVID-cases. In COVID-patients, the 30-day mortality rate was highest at the beginning of the first wave (29% in March-April 2020), reached 17% at the second wave peak (November-December) followed by 11–13% in the third wave (March-July 2021). The mortality in non-COVID geriatric patients showed a similar trend, but of lower magnitude (5–10%). During the incidence peaks, COVID-19 hospitalizations displaced non-COVID geriatric patients.

**Interpretation:**

Hospital admissions and 30-day mortality after hospitalizations for COVID-19 increased in periods of high community transmission, albeit with decreasing mortality rates from wave 1 to 3, with a probable vaccination effect in wave 3. Thus, the healthcare system could not compensate for the high community spread of COVID-19 during the pandemic peaks, which also led to displacing care for non-COVID geriatric patients.

## Introduction

During the first wave of the COVID-19 pandemic in Stockholm, from March to July 2020, mortality for COVID-19 patients hospitalized in geriatric clinics decreased over time [1]. It was unclear at the time whether this was due to improved care or to patient selection of more severe cases earlier in the pandemic due to higher community incidence and an overburdened healthcare system. Our results from that first wave included only in-hospital mortality and did not explore how care for conditions other than COVID-19 was impacted by the pandemic. Age is among the strongest risk factors for COVID-19 and the infection fatality ratio after age 70 is substantial [2,3]. Here, we present data on geriatric hospitalizations for COVID-19 and other causes, including 30-day mortality from admission and compare these to the incidence of COVID-19 and 30-day mortality from COVID-19 in the Stockholm region for persons 70+. We aim to describe how COVID-19 peaks affected geriatric patients 70 and older hospitalized both for COVID-19 and for other causes in Stockholm [4–7].

In previous reports, COVID-19 mortality in hospitalized patients in Sweden increased within each pandemic wave and decreased between pandemic peaks [8,9]. According to the Swedish Board of Health and Welfare, 60-day mortality after admission with COVID-19 decreased from a first-wave peak in March 2020 (25%) to a first nadir in August-September 2020 (10%), then rose again but to lower levels as the second wave progressed to a peak in December 2020 (20%), decreasing steadily afterwards [10]. Decreasing mortality during the first pandemic wave was also observed in studies from the US [11], with subsequent increases in mortality during pandemic surges [12]. A Spanish study reported that COVID-19 patients in the first wave were older, with more comorbidities and higher mortality than in the second wave [13]. However, information is missing on mortality trends later in the pandemic, trends in older population and in geriatric patients with diagnoses other than COVID-19.

The aim of this study is to describe the changes over time in 30 day-mortality after admission of patients 70 years and older, hospitalized in 9 geriatric clinics in the Stockholm region from March 2020 to July 2021. We describe 30-day mortality both for patients hospitalized for COVID-19 and other causes over the three waves of the pandemic. Weekly hospitalizations for COVID-19 and other causes are presented and compared to the average weekly hospitalizations in the same geriatric clinics in 2019. These results are presented in the context of COVID-19 incidence and 30-day mortality and weekly hospitalizations from any cause in population 70 and over in the Stockholm region. 30-day mortality risk relative to date of hospitalization was calculated with logistic regression adjusting for patient characteristics and medications.

## Methods

### Setting

The first COVID-19 case was identified in Stockholm in the end of February 2020. Following the Stockholm region medical catastrophe preparedness plan, a special regional crisis leadership was activated. The geriatric clinics in Stockholm were charged with reorganizing to provide care to persons with COVID-19 and supporting the hospital to ensure continued care of other patients. During the pandemic, the geriatric clinics covered between 12 and 58% of all hospitalized COVID-19 cases in Stockholm [14].

Initially, testing capacity was limited. In April 2020, testing for COVID-19 was prioritized to patients requiring hospitalization and personnel within health and elderly care with suspected COVID-19 symptoms. In May 2020, a recommendation was published for generous testing in nursing homes, including testing before admission. In June 2020 this was extended to other homes, individuals living at home with homecare support and medical care at home, and private individuals. Private individuals could themselves request testing even if they didn’t require hospitalization [15]. Testing for the public occurred at home or in drive-through stations. All hospitalized patients continued to be tested upon admission and could be retested if symptoms occurred or in the context of contact tracing.

Bed availability was a concern and other wards were converted to COVID-19. Personnel was redirected from primary care, other specialist clinics, staffing companies, and through recruitment of temporary personnel. As pressure on the ICU increased, infectious units and geriatrics took on sicker patients, including those needing high flow oxygen. Intensive care in Sweden already experienced pressure before the pandemic and the Stockholm region was the first to experience an explosive increase in ICU hospitalizations during the first pandemic wave. The number of ICU patient-days doubled during April 2020. A campaign hospital was built in the Stockholm International Fair in Älvsjö to increase both hospital and intensive care capacity, but it was never used [16].

### Study population

**Fig 1:**
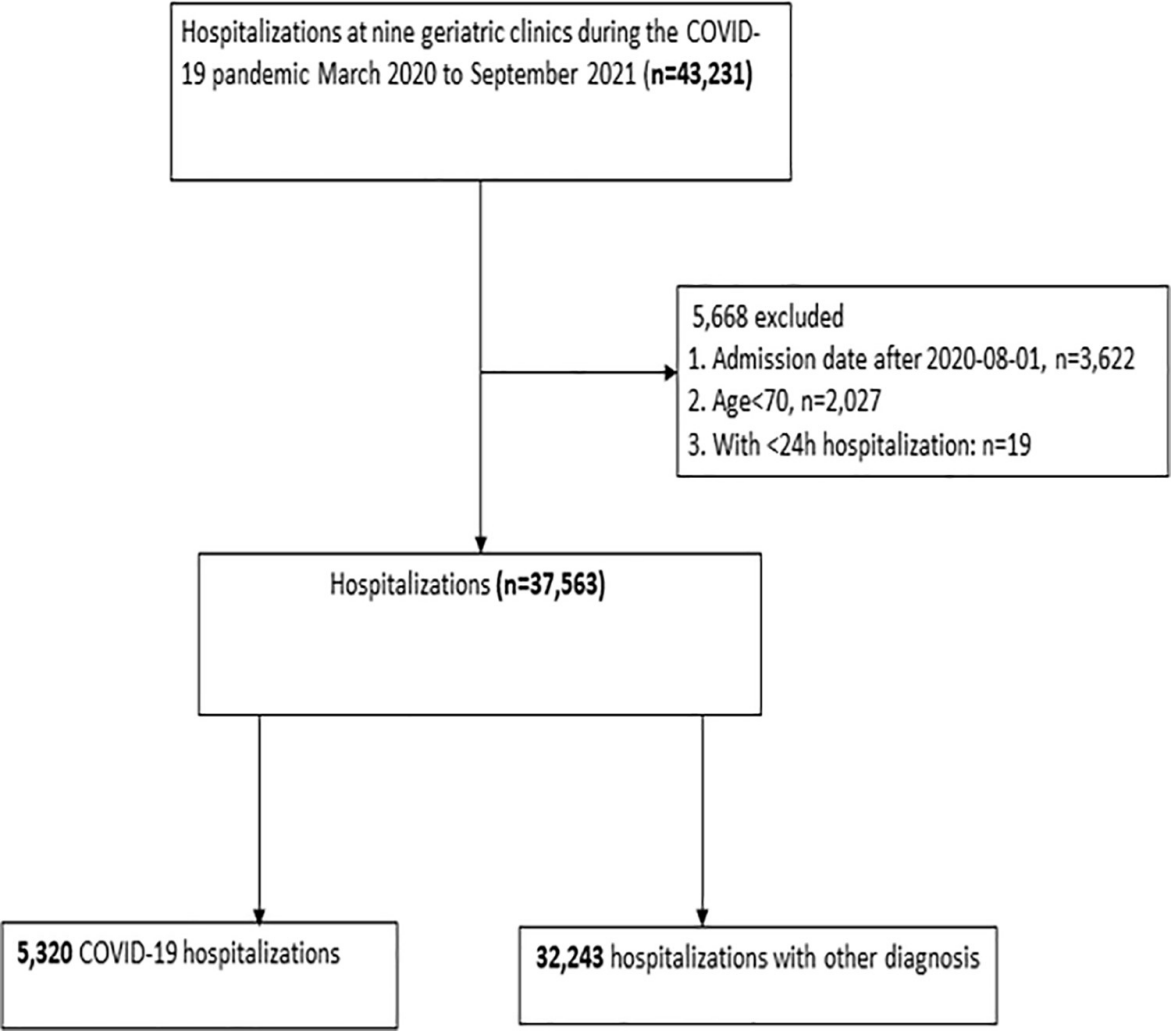
Case selection flow chart.

In the Stockholm region, geriatric inpatient clinics treat and care for patients who are biologically aged and require inpatient geriatric care with specialists in geriatric medicine and multidisciplinary teamwork. Nine out of eleven geriatric hospitals in the Stockholm region agreed to participate in this study. We identified all hospitalizations of patients 70 years and older who were admitted to nine geriatric hospitals in Stockholm, Sweden, from March 6^th^, 2020, to July 31st, 2021. We excluded hospitalizations with a duration less than 24 hours or with an admission date after August 1^st^, 2021, to allow one month follow-up for mortality. A total of 5,320 hospitalizations for COVID-19 were included, together with 32,243 hospitalizations with non-COVID-19 diagnoses during the same period (**Fig 1**). This corresponded to 4,565 individual COVID-19 patients and 19,308 non-COVID-19 patients.

### Exposure and outcome

The study exposure was the hospital admission date (March 1^st^, 2020, to July 31st, 2021). The study outcome was 30-day mortality from admission, irrespective of place of death (in-hospital or post discharge). Patients were censored at death, or the end of follow-up (September 10th, 2021) whichever came first.

### COVID-19 diagnosis and covariates

The diagnosis of COVID-19 followed clinical practice and was based on a positive reverse transcriptase-polymerase chain reaction (RT-PCR) analysis from nasopharyngeal swabs or, in case of a negative RT-PCR, typical clinical picture (including a consultation with a specialist in infectious diseases) and a CT scan with typical COVID-19 findings. Patients with detected COVID-19 regardless of admission, discharge, or at death were considered COVID admissions. All hospitalizations were included, and patients could have multiple hospitalizations. Hospitalizations for diagnoses other than COVID-19 to the same nine geriatric hospitals in Stockholm during the same period comprised the non-COVID group. We collected information on patient demographics, initial vital signs, medications, diagnoses at discharge, and 30-day mortality through the hospital electronic health records. Medications were defined as medications that were present on the patient’s electronic dispensing lists within 24-hours of admission. 30-day mortality was defined from the date of admission to geriatrics. Comorbidities were identified by the other diagnoses present in discharge records. Prior hospitalizations from before the pandemic were not available.

### COVID-19 incidence and mortality in the population 70 years and older in Stockholm

Information on confirmed cases with COVID-19 and mortality data in the population 70 and over in Stockholm was provided by the Swedish Board of Health and Welfare for research purposes (www.socialstyrelsen.se). Incidence data represents detected COVID-19 infections. Thirty-day mortality corresponds to deaths where COVID-19 was the main cause of death within 30 days of a positive test for persons 70 and over in the Stockholm region. Weekly hospitalizations for any cause in this population were also included as reference.

### Weekly hospitalizations in 2019

For comparison, the average weekly hospitalizations registered in 2019 in the 9 geriatric clinics was presented. This was calculated by adding the total number of hospitalizations from all clinics (30,969) and dividing by 52 calendar weeks resulting in an average of 596 new hospitalizations per week.

### Analysis

Summary statistics are displayed as mean ± standard deviation (SD) or median (interquartile range, IQR) or proportions.

30-day mortality rate is graphically presented in relationship to number of new admissions per week and COVID-19 incidence and mortality in the Stockholm region. Via logistic regression models, we assessed the effect of admission date on 30-day mortality, adjusted by age, sex, and medical treatment. Results were reported as odds ratios (OR) and 95% confidence intervals (CI). Mortality rates were also compared against non-COVID-19 patients admitted to the same geriatric clinics during the same period.

All analyses were performed using R (https://www.r-project.org) and Stata version 17.0 (StataCorp, College Station, TX).

### Ethical statement

The Swedish Ethical Review Authority approved the study (Dnr 2020–02146, and 2020–03345). Data were extracted anonymized from electronic records. The Swedish Ethical Review Authority waived patient consent for this study.

## Results

### Characteristics and treatments at admission

**Fig 1** shows the patient selection flowchart. A total of 43,231 hospitalizations (representing 27,257 patients) in the geriatric hospitals were registered. After applying exclusion criteria, 37,563 hospitalizations remained; 5,320 hospitalizations due to COVID-19 and 32,243 hospitalizations due to other causes from March 1st, 2020, to July 31st, 2021. Among the hospitalizations due to COVID-19, 87% (4645 hospitalizations) had positive COVID-19 RT-PCR while 675 (13%) had a negative RT-PCR but fulfilled the clinical and radiological criteria for COVID-19. Patients hospitalized for COVID-19 had a median age of 84 (IQR 78–89) years, 53% were women and 6% of the patients had low saturation (<90%) at admission. During the study period non-vitamin-K antagonist oral anticoagulants (NOACs) were used in 33%, low-molecular-weight heparin (LMWH) in 51%, warfarin in 7% and glucocorticoids in 28% of COVID-19 patients within one day of admission. The median duration of hospitalization was 9 days (IQR 6–13) (**Tables 1 and S1**). The median age of geriatric hospitalizations for other (non-COVID-19) diagnoses was higher (85 IQR 79–90), and their hospital stays were shorter (6 days: IQR 4–9). Compared to non-COVID patients, admission of COVID-19 patients during the pandemic had in general a higher proportion of comorbidities in their discharge records, including hypertension, diabetes, COPD, and asthma, but a lower prevalence of congestive heart failure, cancer and stroke, and lower initial oxygen saturation level.

**Table 1:** Hospitalizations for COVID-19 and other causes in patients 70 and over in geriatric clinics.

	2020–2021		2020										2021					
	Overall		Mar-Apr		May-June		July-Aug		Sep-Oct		Nov-Dec		Jan-Feb		Mar-Apr		May-June-July	
	COVID-19	Non-COVID	COVID-19	Non-COVID	COVID-19	Non-COVID	COVID-19	Non-COVID	COVID-19	Non-COVID	COVID-19	Non-COVID	COVID-19	Non-COVID	COVID-19	Non-COVID	COVID-19	Non-COVID
**N**	5320	32243	990	3177	813	3198	117	4191	118	4736	1329	2770	838	3217	870	4028	245	6926
**Age, year**	84.0 (78.0, 89.0)	85.0 (79.0, 90.0)	84.5 (79.0, 90.0)	86.0 (80.0, 91.0)	85.0 (79.0, 91.0)	86.0 (80.0, 91.0)	86.0 (81.0, 90.0)	85.0 (79.0, 90.0)	85.5 (81.0, 91.0)	85.0 (79.0, 90.0)	84.0 (79.0, 89.0)	85.0 (80.0, 90.0)	83.0 (78.0, 89.0)	85.0 (79.0, 90.0)	81.0 (76.0, 86.0)	85.0 (79.0, 90.0)	81.0 (75.0, 88.0)	85.0 (79.0, 90.0)
**Women**	2822 (53.0%)	19336 (60.0%)	535 (54.0%)	1895 (59.6%)	442 (54.4%)	1900 (59.4%)	63 (53.8%)	2574 (61.4%)	70 (59.3%)	2822 (59.6%)	723 (54.4%)	1635 (59.0%)	429 (51.2%)	1907 (59.3%)	436 (50.1%)	2429 (60.3%)	124 (50.6%)	4174 (60.3%)
**Cormobidities**																		
**CCI**	2.0 (0.0, 3.0)	1.0 (0.0, 3.0)	2.0 (0.0, 3.0)	2.0 (0.0, 3.0)	2.0 (0.0, 3.0)	1.0 (0.0, 3.0)	1.0 (0.0, 3.0)	1.0 (0.0, 3.0)	1.0 (0.0, 3.0)	1.0 (0.0, 3.0)	2.0 (0.0, 3.0)	1.0 (0.0, 3.0)	2.0 (0.0, 3.0)	1.0 (0.0, 3.0)	2.0 (0.0, 3.0)	1.0 (0.0, 3.0)	2.0 (0.0, 3.0)	1.0 (0.0, 3.0)
**Hypertension**	1777 (33.4%)	9804 (30.4%)	380 (38.4%)	1150 (36.2%)	310 (38.1%)	1050 (32.8%)	25 (21.4%)	1305 (31.1%)	43 (36.4%)	1388 (29.3%)	383 (28.8%)	817 (29.5%)	268 (32.0%)	930 (28.9%)	306 (35.2%)	1138 (28.3%)	62 (25.3%)	2026 (29.3%)
**Diabetes**	2014 (37.9%)	9080 (28.2%)	344 (34.7%)	908 (28.6%)	287 (35.3%)	844 (26.4%)	33 (28.2%)	1174 (28.0%)	32 (27.1%)	1360 (28.7%)	510 (38.4%)	769 (27.8%)	331 (39.5%)	887 (27.6%)	367 (42.2%)	1147 (28.5%)	110 (44.9%)	1991 (28.7%)
**Chronic heart failure**	761 (14.3%)	5001 (15.5%)	179 (18.1%)	668 (21.0%)	152 (18.7%)	541 (16.9%)	11 (9.4%)	627 (15.0%)	21 (17.8%)	680 (14.4%)	176 (13.2%)	430 (15.5%)	115 (13.7%)	480 (14.9%)	85 (9.8%)	622 (15.4%)	22 (9.0%)	953 (13.8%)
**Myocardial Infarction**	233 (4.4%)	1206 (3.7%)	43 (4.3%)	160 (5.0%)	31 (3.8%)	123 (3.8%)	1 (0.9%)	163 (3.9%)	4 (3.4%)	162 (3.4%)	57 (4.3%)	102 (3.7%)	39 (4.7%)	103 (3.2%)	44 (5.1%)	140 (3.5%)	14 (5.7%)	253 (3.7%)
**Chronical pulmonary disease**	709 (13.3%)	3189 (9.9%)	148 (14.9%)	447 (14.1%)	108 (13.3%)	379 (11.9%)	9 (7.7%)	417 (9.9%)	20 (16.9%)	446 (9.4%)	185 (13.9%)	225 (8.1%)	114 (13.6%)	313 (9.7%)	106 (12.2%)	345 (8.6%)	19 (7.8%)	617 (8.9%)
**Asthma**	185 (3.5%)	538 (1.7%)	36 (3.6%)	76 (2.4%)	24 (3.0%)	60 (1.9%)	3 (2.6%)	56 (1.3%)	8 (6.8%)	74 (1.6%)	41 (3.1%)	46 (1.7%)	28 (3.3%)	48 (1.5%)	39 (4.5%)	70 (1.7%)	6 (2.4%)	108 (1.6%)
**Cancer**	331 (6.2%)	2173 (6.7%)	74 (7.5%)	266 (8.4%)	49 (6.0%)	246 (7.7%)	16 (13.7%)	275 (6.6%)	10 (8.5%)	289 (6.1%)	84 (6.3%)	186 (6.7%)	41 (4.9%)	219 (6.8%)	42 (4.8%)	266 (6.6%)	15 (6.1%)	426 (6.2%)
**Stroke**	307 (5.8%)	2254 (7.0%)	66 (6.7%)	257 (8.1%)	58 (7.1%)	289 (9.0%)	4 (3.4%)	316 (7.5%)	9 (7.6%)	327 (6.9%)	79 (5.9%)	188 (6.8%)	38 (4.5%)	224 (7.0%)	39 (4.5%)	282 (7.0%)	14 (5.7%)	371 (5.4%)
**Atrial fibrillation**	1061 (19.9%)	6578 (20.4%)	217 (21.9%)	896 (28.2%)	195 (24.0%)	765 (23.9%)	25 (21.4%)	851 (20.3%)	26 (22.0%)	942 (19.9%)	273 (20.5%)	557 (20.1%)	151 (18.0%)	638 (19.8%)	139 (16.0%)	714 (17.7%)	35 (14.3%)	1215 (17.5%)
**Initial S02<90%**	342 (6.4%)	991 (3.1%)	89 (9.0%)	135 (4.2%)	39 (4.8%)	100 (3.1%)	3 (2.6%)	116 (2.8%)	7 (5.9%)	153 (3.2%)	99 (7.4%)	90 (3.2%)	50 (6.0%)	87 (2.7%)	42 (4.8%)	134 (3.3%)	13 (5.3%)	176 (2.5%)
**Medications**																		
**ACEI**	1229 (23.1%)	7445 (23.1%)	246 (24.8%)	745 (23.4%)	198 (24.4%)	772 (24.1%)	21 (17.9%)	924 (22.0%)	24 (20.3%)	1049 (22.1%)	276 (20.8%)	673 (24.3%)	204 (24.3%)	768 (23.9%)	190 (21.8%)	940 (23.3%)	70 (28.6%)	1574 (22.7%)
**ARB**	1462 (27.5%)	8602 (26.7%)	251 (25.4%)	803 (25.3%)	209 (25.7%)	836 (26.1%)	32 (27.4%)	1120 (26.7%)	40 (33.9%)	1274 (26.9%)	378 (28.4%)	737 (26.6%)	243 (29.0%)	805 (25.0%)	248 (28.5%)	1084 (26.9%)	61 (24.9%)	1943 (28.1%)
**β-blocker**	2745 (51.6%)	17298 (53.6%)	531 (53.6%)	1744 (54.9%)	419 (51.5%)	1720 (53.8%)	65 (55.6%)	2231 (53.2%)	54 (45.8%)	2515 (53.1%)	681 (51.2%)	1445 (52.2%)	436 (52.0%)	1691 (52.6%)	430 (49.4%)	2202 (54.7%)	129 (52.7%)	3750 (54.1%)
**CCB**	1621 (30.5%)	9828 (30.5%)	295 (29.8%)	947 (29.8%)	237 (29.2%)	955 (29.9%)	34 (29.1%)	1240 (29.6%)	24 (20.3%)	1404 (29.6%)	412 (31.0%)	847 (30.6%)	255 (30.4%)	1018 (31.6%)	286 (32.9%)	1245 (30.9%)	78 (31.8%)	2172 (31.4%)
**Diuretics**	2819 (53.0%)	17667 (54.8%)	563 (56.9%)	1740 (54.8%)	449 (55.2%)	1746 (54.6%)	66 (56.4%)	2229 (53.2%)	74 (62.7%)	2562 (54.1%)	672 (50.6%)	1475 (53.2%)	453 (54.1%)	1745 (54.2%)	410 (47.1%)	2275 (56.5%)	132 (53.9%)	3895 (56.2%)
**Statins**	2148 (40.4%)	12580 (39.0%)	380 (38.4%)	1181 (37.2%)	309 (38.0%)	1272 (39.8%)	40 (34.2%)	1541 (36.8%)	48 (40.7%)	1810 (38.2%)	548 (41.2%)	1077 (38.9%)	354 (42.2%)	1328 (41.3%)	380 (43.7%)	1569 (39.0%)	89 (36.3%)	2802 (40.5%)
**Warfarin**	388 (7.3%)	2440 (7.6%)	85 (8.6%)	282 (8.9%)	61 (7.5%)	252 (7.9%)	15 (12.8%)	302 (7.2%)	7 (5.9%)	361 (7.6%)	93 (7.0%)	188 (6.8%)	62 (7.4%)	283 (8.8%)	48 (5.5%)	312 (7.7%)	17 (6.9%)	460 (6.6%)
**LMWH**	2732 (51.4%)	5960 (18.5%)	410 (41.4%)	622 (19.6%)	427 (52.5%)	598 (18.7%)	39 (33.3%)	786 (18.8%)	59 (50.0%)	798 (16.8%)	772 (58.1%)	584 (21.1%)	417 (49.8%)	643 (20.0%)	509 (58.5%)	783 (19.4%)	99 (40.4%)	1146 (16.5%)
**NOAC**	1745 (32.8%)	9975 (30.9%)	262 (26.5%)	942 (29.7%)	285 (35.1%)	1012 (31.6%)	41 (35.0%)	1245 (29.7%)	34 (28.8%)	1463 (30.9%)	429 (32.3%)	846 (30.5%)	315 (37.6%)	959 (29.8%)	287 (33.0%)	1245 (30.9%)	92 (37.6%)	2263 (32.7%)
**Glucocorticoids**	1492 (28.0%)	5426 (16.8%)	151 (15.3%)	585 (18.4%)	148 (18.2%)	515 (16.1%)	23 (19.7%)	675 (16.1%)	31 (26.3%)	802 (16.9%)	465 (35.0%)	439 (15.8%)	250 (29.8%)	521 (16.2%)	338 (38.9%)	669 (16.6%)	86 (35.1%)	1220 (17.6%)
**Antibiotics**	1798 (33.8%)	11154 (34.6%)	369 (37.3%)	1071 (33.7%)	252 (31.0%)	1000 (31.3%)	41 (35.0%)	1360 (32.5%)	41 (34.7%)	1547 (32.7%)	426 (32.1%)	986 (35.6%)	280 (33.4%)	1111 (34.5%)	288 (33.1%)	1469 (36.5%)	101 (41.2%)	2610 (37.7%)
**Follow up**																		
**Days of hospitalization**	9.0 (6.0, 13.0)	6.0 (4.0, 9.0)	9.0 (6.0, 14.0)	6.0 (4.0, 8.0)	9.0 (6.0, 14.0)	7.0 (4.0, 9.0)	9.0 (6.0, 13.0)	7.0 (4.0, 9.0)	11.0 (8.0, 19.0)	6.0 (4.0, 9.0)	9.0 (6.0, 14.0)	7.0 (5.0, 9.0)	8.0 (6.0, 12.0)	7.0 (5.0, 9.0)	7.0 (5.0, 11.0)	7.0 (4.0, 9.0)	8.0 (5.0, 10.0)	6.0 (4.0, 8.0)
**30-day mortality**	910 (17.1%)	2022 (6.3%)	282 (28.5%)	309 (9.7%)	134 (16.5%)	199 (6.2%)	8 (6.8%)	263 (6.3%)	21 (17.8%)	225 (4.8%)	228 (17.2%)	193 (7.0%)	106 (12.6%)	209 (6.5%)	99 (11.4%)	231 (5.7%)	32 (13.1%)	393 (5.7%)

Abbreviations: S02<90% peripheral oxygen saturation under 90%; ACEI, angiotensin-converting enzyme inhibitors; ARB, angiotensin receptor blockers; CCB, calcium channel blockers; LMWH, low-molecular-weight heparin; NOAC, Non-vitamin-K antagonist oral anticoagulant. Days of hospitalization: median number of days hospitalized (interquartile range). 30-day mortality is calculated from the date of hospitalization.

**Fig 2:**
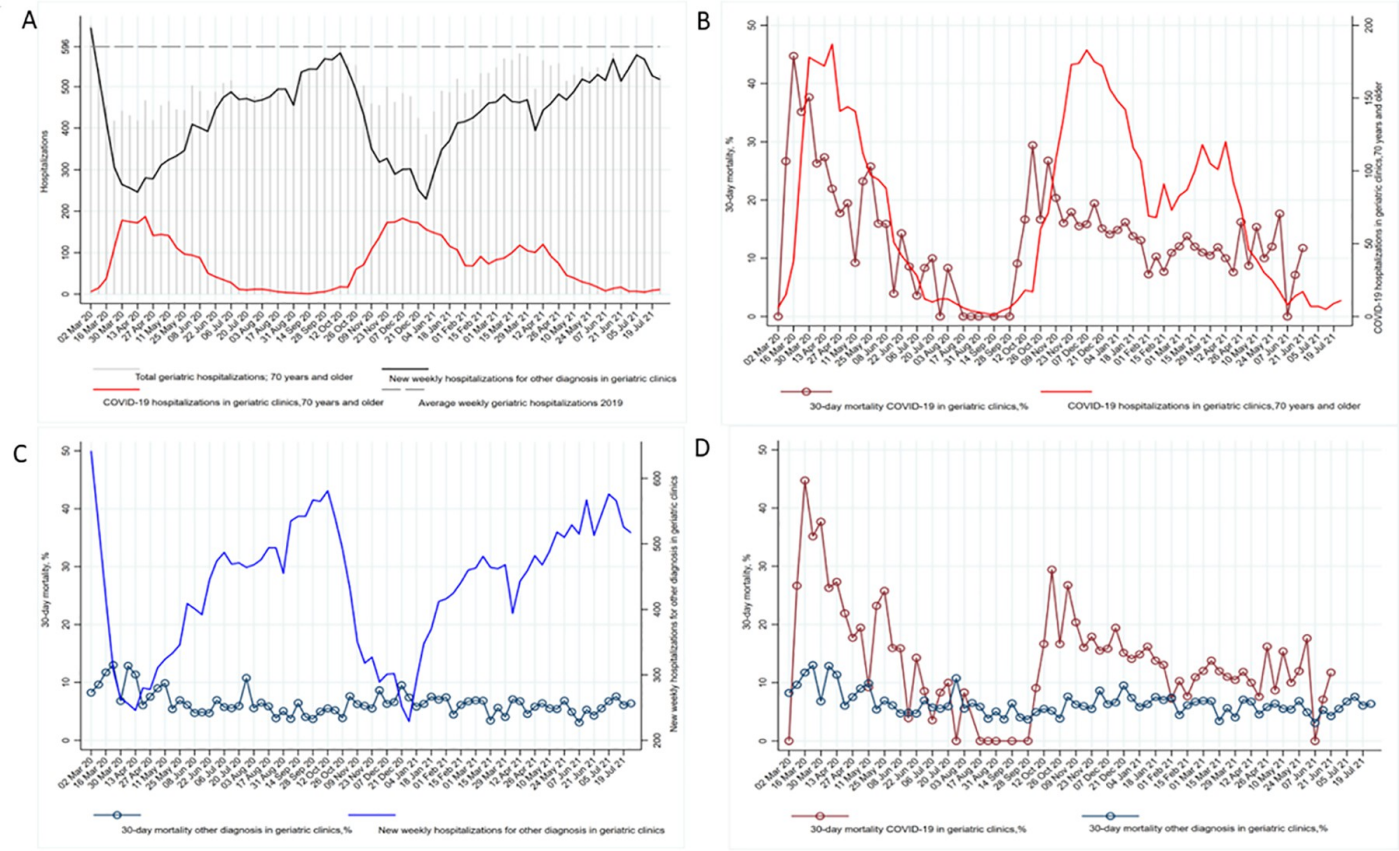
Hospitalizations and mortality throughout the COVID-19 pandemic for persons aged 70 and above: A) Number of weekly hospitalizations of patients 70 and above in nine geriatric clinics in Stockholm from March 2020 to July 2021; B) 30-day COVID-19 mortality in relationship to weekly hospitalizations in geriatric clinics in patients 70 and above; C) 30-day mortality and hospitalizations for non-COVID causes in geriatric clinics for patients 70 and over; D) 30-day COVID-19 mortality and Non-COVID cause mortality.

The total number of hospitalizations in the geriatric hospitals fell sharply at the beginning of the pandemic from a pre-pandemic weekly average of 596 in 2019. After this initial drop in the first wave, geriatric hospitalizations rallied, averaging 507 hospitalizations per week but without a sustained return to pre-pandemic levels. When COVID-19 cases increased, non-COVID-19 cases decreased accordingly (**Fig 2A**; **S2 Table**).

### Thirty-day mortality

Thirty-day mortality was highest at the beginning of the first wave (29% in March-April 2020 for COVID-19; 10% for non-COVID-19), decreased as the first wave subsided (7% July-August for COVID-19; 6% for non-COVID-19), and increased again for COVID cases in the second wave (17% November-December for COVID; 7% for non-COVID). Thirty-day mortality remained more stable in the third wave (11 to 13% March-July 2021 for COVID-19; 6% non-COVID-19) (**Tables 1 and S1**).

**Fig 2B** shows the relationship between weekly COVID-19 hospital admissions to the nine geriatric hospitals and 30-day mortality. Mortality is lowest when few COVID-19 patients are hospitalized. **Fig 2C** presents the same relationship but for other geriatric patients. The lowest mortality rates appear in the interpandemic peaks, when COVID hospitalizations were lowest and non-COVID hospitalizations were highest. **Fig 2D** compared 30-day mortality in COVID-19 patients and non-COVID causes. In general, mortality in COVID-19 varied over time, mortality in non-COVID was flatter.

**Fig 3:**
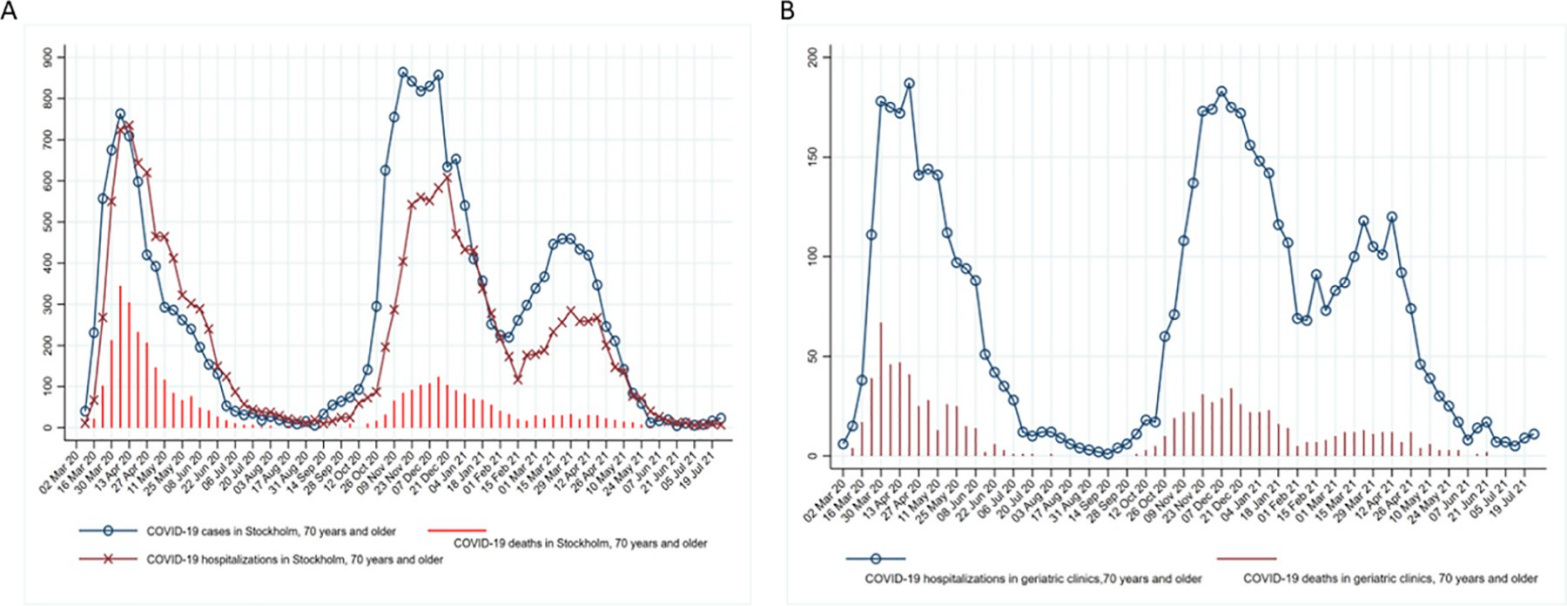
Relationship between confirmed cases of COVID-19, hospitalizations, and deaths in A) Stockholm B) Geriatric clinics.

**Fig 3** shows the relationship between total COVID-19 cases and deaths in the Stockholm region and COVID-19 hospitalizations and deaths in the geriatric hospitals. The number of hospitalizations and the 30-day mortality rates increased with each pandemic peak and decreased between the peaks. The 30-day mortality rate after a positive test in Stockholm and the 30-day mortality rate after admission in the geriatric hospitals followed the pandemic and hospitalization curves. The smaller increase in the third wave probably indicates a vaccination effect (**Fig 3A and 3B**).

**Table 2** shows 30-day mortality risk from logistic regression, adjusted by age, sex, Charlson Comorbidity Index (CCI) and treatment. Compared with patients admitted to geriatrics for COVID-19 in November-December 2020, the risk of 30-day mortality was higher (OR 1.92, 95% CI 1.55–2.37) in March-April 2020, and lower in July August 2020 (OR 0.31, 0.15–0.66). A downward tendency appeared again in January-February 2021(OR 0.70, 0.54–0.91), March-April 2021 (OR 0.72, 0.55–0.94) and May-June-July (OR 0.75, 0.49–1.13) (**Table 2**). Older age, male sex, high number of comorbidities, and the use of β-blockers, diuretics and antibiotics were associated with a higher death risk; in contrast, the use of calcium channel blockers (CCB) and statins were associated with a lower risk of death (**Table 2**).

**Table 2:** Odds ratios for 30-day mortality in geriatric patients with COVID-19 and other diagnoses throughout the pandemic.

	COVID-19		Non-COVID	
	OR	95%CI	OR	95%CI
**Mar-Apr 2020**	1.92***	1.55,2.37	1.33**	1.10,1.61
**May-June 2020**	0.91	0.72,1.17	0.85	0.69,1.05
**July-Aug 2020**	0.31**	0.15,0.66	0.90	0.74,1.09
**Sep-Oct 2020**	0.99	0.59,1.64	0.67***	0.55,0.82
**Nov-Dec 2020**	ref		ref	
**Jan-Feb 2021**	0.70**	0.54,0.91	0.93	0.76,1.14
**Mar-Apr 2021**	0.72*	0.55,0.94	0.81*	0.67,0.99
**May-June-July 2021**	0.75	0.49,1.13	0.82*	0.69,0.98
**Age**				
**70–79**	ref		ref	
**80–89**	2.05***	1.68,2.51	1.62***	1.42,1.85
**90+**	3.06***	2.45,3.82	2.42***	2.11,2.77
**Women**	0.63***	0.54,0.74	0.68***	0.62,0.75
**CCI**	1.16***	1.12,1.20	1.18***	1.16,1.21
**ACEI**	0.94	0.78,1.14	0.84**	0.75,0.94
**ARB**	0.84	0.70,1.01	0.72***	0.64,0.80
**β -blocker**	1.20*	1.02,1.41	1.25***	1.13,1.39
**CCB**	0.80*	0.68,0.95	0.72***	0.65,0.80
**Diuretics**	1.32***	1.12,1.55	1.49***	1.34,1.65
**Statins**	0.70***	0.59,0.82	0.74***	0.66,0.82
**Warfarin**	1.15	0.87,1.52	1.06	0.91,1.25
**LMWH**	1.06	0.88,1.26	1.23***	1.10,1.38
**NOAC**	0.93	0.77,1.13	0.98	0.88,1.08
**Glucocorticoids**	1.22*	1.03,1.45	1.18**	1.05,1.33
**Antibiotic**	1.51***	1.29,1.75	1.27***	1.16,1.40

Abbreviations: ACEI, angiotensin-converting enzyme inhibitors; ARB, angiotensin receptor blockers; CCB, calcium channel blockers; LMWH, low-molecular-weight heparin; NOAC, Non-vitamin-K antagonist oral anticoagulant.

### Thirty-day mortality in non-COVID-19 hospitalizations

Compared with hospitalizations in November-December 2020, hospitalizations in March-April 2020 had the highest mortality risk (OR 1.33; 95 CI 1.10–1.61), while hospitalizations from September-October 2020 (OR 0.67; 95% CI 0.55–0.82), March-April 2021 (0.81; 95% CI 0.67–0.99) and May-July 2021 (OR 0.82; 95% CI 0.69–0.98) presented lower risk, after adjusting (**Tables 1 and S1 and Fig 2**).

## Discussion

Several important observations appear in this study of hospitalized geriatric patients in Stockholm, Sweden. First, hospital admissions fell sharply at the beginning of the pandemic and didn’t reach pre-pandemic levels throughout the whole observation period. Second, 30-day mortality rose and fell with the pandemic waves. This connection between the epidemiological situation in the community and in-hospital mortality was compelling for patients hospitalized for COVID-19 but appeared also in geriatric patients hospitalized for other reasons. Both external factors, and factors related to hospital organization and care explain our findings.

### Factors external to hospital care and organization

As shown in Fig 3, there was a close relationship between epidemic peaks and geriatric hospitalizations and deaths, showing that focused protection of the elderly failed [17] Focused protection may have, however, reduced the number of beds needed for other conditions: hip and other osteoporotic fractures decreased in the 70+ population, possibly due to the recommendations for this age group to avoid social contact, which limited going out [18].

Care seeking behaviour and the diagnostic capacity of the healthcare system was affected: for example, the Swedish Stroke Register reports a decrease in the number of detected strokes in 2020 and 2021 compared to 2019 [19], while the Swedish registry for cognitive/dementia disorders reported a 30% reduction in diagnostic work-ups [20]. One US study included 23 million emergency department (ED) visits for elderly Medicare beneficiaries, and showed a drop in ED visits, together with increased admission rate and mortality suggesting that patients avoided seeking care, which selected a sicker patient population into the ED [21]. Other studies found a decrease in ED visits for cardiovascular conditions early in the pandemic [22–24]. In contrast to those studies, we saw a drop in hospitalizations in our cohort but not a complete recovery afterwards. We do not have data on ED visits so we cannot distinguish between patient care-seeking behaviours and triage inside the ED. As seen in Fig 2A COVID-19 patients displaced other diagnoses from geriatric care.

The mortality peak in geriatric clinics closely followed the pandemic waves but was higher in the first than in the second wave. This was more evident for COVID-19 patients but appeared also in non-COVID geriatric patients in multivariate regression analysis. Several studies, including ours, have noted a decrease in mortality throughout the first wave of the pandemic [1,25]. A subsequent increase was observed again during the second wave in Sweden [9,26]. COVID-19 incidence was more severely underreported in the first wave in Sweden than later in the pandemic, and may partially explain the higher mortality by selecting for sicker patients [26]. Some patients were treated in their nursing homes and never transferred to hospital and most patients admitted to inpatient geriatric hospital care in our cohort lived in their own homes prior to admission. As a proxy for disease severity in geriatric hospitalizations, the proportion of patients presenting with oxygen saturation under 90% at baseline decreased from 8.9% in March-April 2020 to 2% in June, increasing again to 7.7% in December 2020. In our previous article we showed that low oxygen saturation at baseline explained some, but not all, of the temporal trend in mortality observed during the first wave.(1). In an international context, studies from Africa [27], Italy [28], Germany [29] and raw case fatality ratio (CFR) in 53 countries [30] also showed a significant difference in COVID-mortality between the first and the second wave.

Finally, vaccinations changed the association between incidence and mortality in the third wave and averted the increase in 30-day mortality that had been apparent with increasing hospitalizations in the previous waves. The COVID-19 vaccination campaign began at the end of December 2020 in nursing homes and its effects on mortality were already felt in March-April 2021, as also seen in our cohort [31].

### Hospital organization and care

Geriatric clinics scaled up during the pandemic, opening more beds and receiving resources from other clinics, particularly elective surgical and diagnostic units. A field hospital was prepared on the premises of the Stockholm International Fair in Älvsjö but never opened. Personnel resources (particularly nurses) were the limiting factor to opening and maintaining more geriatric beds. Patients and personnel were transferred between hospitals but this couldn’t affect the total capacity of the Stockholm region. Despite this effort, the total number of hospitalizations fell in the beginning of the pandemic and didn’t recuperate. Longer hospital stays for COVID-19 patients (median 9 vs 6 days in non-COVID-19; Table 1) could explain this finding. This longer length of hospitalization for COVID-19 patients is in line with previous international reports and can be considered a characteristic of the disease [11,32]. The higher care burden for COVID-19 patients, together with the time required to don and doff protective equipment, and need for separation of COVID-19 and non-COVID cohorts, could have led to lower total number of hospital stays. Furthermore, a reduction in elective procedures also contributed to the total fall in admissions in geriatrics. Hospital admissions further decreased during the summer months: the customary reductions in available beds due to staff vacation which occur every summer was compounded by the receding COVID-19 waves. The supply of geriatric hospitalizations in our cohort was less elastic, remained relatively stable during the pandemic and was lower than in 2019. Triage was probably harsher in the first wave leading to a sicker patient population and higher mortality. Medical care and guidelines changed throughout the pandemic and may have reduced mortality starting in the second wave. A similar pattern of increasing hospitalization days per patient and decreasing total hospitalizations was also seen in intensive care [16].

It should be noted that the 30- day mortality rate for COVID-19 patients reported in our study is based on a large cohort of older people in geriatric clinics in Stockholm, excluding nursing home patients treated in place, mild cases treated at home or cases treated in ICU or infectious disease wards instead of the geriatric clinics. Second, during the first wave of pandemic, the treatment and care of COVID-19 patients underwent changes (https://www.internetmedicin.se/behandlingsoversikter/infektion/covid-19/), and these improvements are likely to be part of the reason for the decline in mortality during the first wave. In the second wave, the national guidelines for hospitalized COVID-19 patients didn’t undergo major changes. In a previous report, we have shown that the prognosis of COVID-19 for the most frail during the first wave was ominous [33]. It is possible that the most frail died during the first wave, contributing to lower mortality during the second and successive waves.

We believe that due to the inelasticity of hospitalizations, increasing COVID-19 admissions during pandemic waves displaced non-COVID admissions. This led to more stringent selection of admissions, a sicker patient population and greater overall mortality. The displacement of milder cases during pandemic waves is worrying. Changes in treatment guidelines, particularly use of anticoagulants and corticosteroids led to improvement in the outcome of patients with COVID-19 [34]. However, except for vaccines and the third wave, these changes were implemented relatively early in the pandemic (before or during the summer of 2020) and cannot explain all the later changes in mortality [34]. Our data is limited in the ascertainment of comorbidities and treatments during hospitalization which precludes further speculation. A decrease in the quality of care during the pandemic peaks due to greater pressure on the healthcare system cannot be excluded.

### Implications for future crises and resource planing

There are several lessons to be drawn from the pandemic. Other countries implemented costly lock-downs. The Corona Commission (launched by the Swedish government to independently evaluate the management of the pandemic) determined that the principle of non-mandatory measures was sound, but that stronger and quicker interventions would have been possible within this voluntary framework [35]. The Corona Commission also determined that focused protection failed to safeguard the elderly and frail and that single factor most likely responsible for the high death toll in nursing homes was the high epidemic spread of the virus in society [17]. Our study, which mostly included older persons living at home, also showed high agreement between community spread and geriatric hospitalizations and mortality.

In Sweden, the governing principle is that the organization in charge of an activity under normal circumstances is also responsible for management in times of crisis. Previous crises have shown that this principle can cause delayed response because actors who are not experts in crisis management wait too long out of fear of taking measures on incomplete information [36]. In the highly fragmented Swedish care and healthcare system, there was a lack of national overview [36]. As seen by the wasted investment in the field hospital in Älvsjö, the bottleneck in hospital capacity was personnel, not physical beds. In Sweden, hospital beds per capita have been consistently falling for decades [37]. Although some of this capacity is substituted by nursing home beds, the average of 2.1 hospital beds per 1000 capita, compared to 8 in Germany or 5.9 in France, may simply be too low [37]. Swedish Regions (which are responsible for public hospitals) can implement crisis agreements which are pre-negotiated with labor unions. These allow for exceptions to maximun labor hours in times of crisis, but require costly overtime compensation. These crisis agreements were applied in some clinics (mainly ICUs). In any case, these can only be short-term solutions since they are costly and necessitate time off for staff to recuperate afterwards.

### Strengths and limitations

Strengths of this study is the large geriatric cohort including nine out of eleven geriatric clinics in Stockholm treating patients with COVID-19. The access to patient-level weekly data, comparison to 2019 admissions and to COVID-19 incidence, hospitalizations, and mortality in the community are novel. Also novel is the analysis of geriatric hospitalizations for other causes and the long study period including three pandemic waves over a period of 17 months. In Sweden, geriatric hospitalizations are indicated on criteria of biological (and not chronological aging). Non-frail and non-comorbid older patients may have been hospitalized in other clinics (eg. infectious diseases) and not included in our cohort. Nine out of eleven existing geriatric clinics in Stockholm participated in the study. Living situation, comorbidities and medications were obtained from electronic health records for the current hospitalization with imperfect ascertainment. Previous comorbidities or hospitalizations were not available. We did not have information on vaccinations, which would have been useful for assessing the decline of mortality in the third wave of the pandemic.

## Conclusion

Thirty-day mortality was highest at the peak of the first wave, decreased in the inter-wave period and then increased again but to a lower peak in the second wave. The mortality increase of the third wave was probably averted by the vaccination campaign. Non-COVID mortality showed a similar trend but with lower magnitude. During COVID-19 incidence peaks, COVID-19 hospitalizations displaced non-COVID geriatric patients. Despite a massive organizational effort and the individual effort and sacrifice of health care workers, the healthcare system could not compensate for high community spread of COVID-19 during the pandemic peaks. Hospital admissions fell at the beginning of the pandemic and never returned to pre-pandemic levels, probably reflecting the greater complexity of COVID-19 patients compared to ordinary geriatric patients. The sustained fall in hospital admissions is particularly worrisome considering the backlog of cancelled and deferred care caused by the pandemic. COVID-19 disproportionally affects geriatric patients, and the broader pandemic has disrupted geriatric care.

## Supporting information

S1 Table Hospitalizations for COVID-19 and other causes in patients 70 years old and over in geriatric clinics. (DOCX)

S2 Table COVID-19 cases, mortality and hospitalizations in Stockholm and in geriatric clinics for the population 70 years old and above. (DOCX)

## References

[1] Xu H, Garcia-Ptacek S, Annetorp M, Cederholm T, Engel G, Engstrom M, et al. Decreased Mortality Over Time During the First Wave in Patients With COVID-19 in Geriatric Care: Data From the Stockholm GeroCovid Study. J Am Med Dir Assoc. 2021;22(8):1565–73 e4. doi: 10.1016/j.jamda.2021.06.005 34216553 PMC8196313

[2] Seoane B. A scaling approach to estimate the age-dependent COVID-19 infection fatality ratio from incomplete data. PLoS One. 2021;16(2):e0246831. doi: 10.1371/journal.pone.0246831 33596249 PMC7888669

[3] Russell TW, Hellewell J, Jarvis CI, van Zandvoort K, Abbott S, Ratnayake R, et al. Estimating the infection and case fatality ratio for coronavirus disease (COVID-19) using age-adjusted data from the outbreak on the Diamond Princess cruise ship, February 2020. Euro Surveill. 2020;25(12). doi: 10.2807/1560-7917.ES.2020.25.12.2000256 32234121 PMC7118348

[4] Horby P, Lim WS, Emberson JR, Mafham M, Bell JL, Linsell L, et al. Dexamethasone in Hospitalized Patients with Covid-19—Preliminary Report. The New England journal of medicine. 2020.

[5] Beigel JH, Tomashek KM, Dodd LE, Mehta AK, Zingman BS, Kalil AC, et al. Remdesivir for the Treatment of Covid-19—Final Report. The New England journal of medicine. 2020. 10.1056/NEJMoa2007764 PMC7262788 32445440

[6] Paolisso P, Bergamaschi L, D’Angelo EC, Donati F, Giannella M, Tedeschi S, et al. Preliminary Experience With Low Molecular Weight Heparin Strategy in COVID-19 Patients. Frontiers in pharmacology. 2020;11:1124. 32848743 10.3389/fphar.2020.01124 PMC7424043

[7] Wang Y, Zhang D, Du G, Du R, Zhao J, Jin Y, et al. Remdesivir in adults with severe COVID-19: a randomised, double-blind, placebo-controlled, multicentre trial. Lancet (London, England). 2020;395(10236):1569–78. doi: 10.1016/S0140-6736(20)31022-9 32423584 PMC7190303

[8] Strålin K, Wahlström E, Walther S, Bennet-Bark AM, Heurgren M, Lindén T, et al. Mortality trends among hospitalised COVID-19 patients in Sweden: A nationwide observational cohort study. Lancet Reg Health Eur. 2021;4:100054. doi: 10.1016/j.lanepe.2021.100054 33997829 PMC7907732

[9] Strålin K, Wahlström E, Walther S, Bennet-Bark AM, Heurgren M, Lindén T, et al. Second wave mortality among patients hospitalised for COVID-19 in Sweden: a nationwide observational cohort study. medRxiv. 2021:2021.03.29.21254557. 10.1016/j.lanepe.2021.100054 PMC7907732 33997829

[10] Welfare SBoHa. Healthcare and Covid. 2021.

[11] Nguyen JL, Benigno M, Malhotra D, Reimbaeva M, Sam Z, Chambers R, et al. Hospitalization and mortality trends among patients with confirmed COVID-19 in the United States, April through August 2020. Journal of Public Health Research. 2021. doi: 10.4081/jphr.2021.2244 34711044 PMC8874841

[12] Moon RC, Mackey RH, Cao Z, Emont S, Schott LL, Gayle J, et al. Is COVID-19 Less Deadly Now?—Trends of In-Hospital Mortality Among Hospitalized COVID-19 Patients in the United States. Clin Infect Dis. 2021. 10.1093/cid/ciab830 PMC8522416 34534276

[13] Aznar-Gimeno R, Pano-Pardo JR, Esteban LM, Labata-Lezaun G, Esquillor-Rodrigo MJ, Lanas A, et al. Changes in severity, mortality, and virus genome among a Spanish cohort of patients hospitalized with SARS-CoV-2. Sci Rep. 2021;11(1):18844. doi: 10.1038/s41598-021-98308-x 34552127 PMC8458298

[14] Uvhagen HSCK, Øvretveit J., Flink M., & Sparring V. Internal, collaborative, and governmental influences on crisis management response in geriatric care during the Covid-19 pandemic–a qualitative interview study. In: Karolinska Institutet S, editor. 2022.

[15] Elderly care during the pandemic. Partial repport of the Corona Commission. The State’s Public Investigations. SOU 2020:80, ISBN 978-91-38-25132-4 ISSN 0375-250X.

[16] Sweden during the pandemic. Volume 2. Healthcare and poublic health. Partial repport of the Corona Commision. The State’s public investigations. SOU 2021:89

[17] Commission C. Summary in English: The elderly care in the pandemic. In: Investigations PG, editor. 2020.

[18] Rydberg EM, Möller M, Ekelund J, Wolf O, Wennergren D. Does the Covid-19 pandemic affect ankle fracture incidence? Moderate decrease in Sweden. Acta Orthop. 2021;92(4):381–4. doi: 10.1080/17453674.2021.1907517 33821759 PMC8381968

[19] Register TSS. Covid-19 report Stroke. 2021.

[20] SveDem Annual report 2021. 2021.

[21] Smulowitz PB, O’Malley AJ, Khidir H, Zaborski L, McWilliams JM, Landon BE. National Trends In ED Visits, Hospital Admissions, And Mortality For Medicare Patients During The COVID-19 Pandemic. Health Affairs. 2021;40(9):1457–64. doi: 10.1377/hlthaff.2021.00561 34495730

[22] Pines JM, Zocchi MS, Black BS, Celedon P, Carlson JN, Moghtaderi A, et al. The effect of the COVID-19 pandemic on emergency department visits for serious cardiovascular conditions. Am J Emerg Med. 2021;47:42–51. doi: 10.1016/j.ajem.2021.03.004 33770713 PMC7939976

[23] De Rosa S, Spaccarotella C, Basso C, Calabro MP, Curcio A, Filardi PP, et al. Reduction of hospitalizations for myocardial infarction in Italy in the COVID-19 era. Eur Heart J. 2020;41(22):2083–8. doi: 10.1093/eurheartj/ehaa409 32412631 PMC7239145

[24] Sokolski M, Gajewski P, Zymlinski R, Biegus J, Berg JMT, Bor W, et al. Impact of Coronavirus Disease 2019 (COVID-19) Outbreak on Acute Admissions at the Emergency and Cardiology Departments Across Europe. Am J Med. 2021;134(4):482–9. doi: 10.1016/j.amjmed.2020.08.043 33010226 PMC7526639

[25] Strålin K, Wahlström E, Walther S, Bennet-Bark AM, Heurgren M, Lindén T, et al. Mortality trends among hospitalised COVID-19 patients in Sweden: A nationwide observational cohort study. The Lancet Regional Health–Europe. 2021;4. doi: 10.1016/j.lanepe.2021.100054 33997829 PMC7907732

[26] Welfare SBoHa. Statistics on hospitalizations of patients with Covid-19. 2021.

[27] Salyer SJ, Maeda J, Sembuche S, Kebede Y, Tshangela A, Moussif M, et al. The first and second waves of the COVID-19 pandemic in Africa: a cross-sectional study. Lancet (London, England). 2021;397(10281):1265–75. doi: 10.1016/S0140-6736(21)00632-2 33773118 PMC8046510

[28] Dorrucci M, Minelli G, Boros S, Manno V, Prati S, Battaglini M, et al. Excess Mortality in Italy During the COVID-19 Pandemic: Assessing the Differences Between the First and the Second Wave, Year 2020. Frontiers in public health. 2021;9:669209. doi: 10.3389/fpubh.2021.669209 34336767 PMC8322580

[29] Gessler N, Gunawardene MA, Wohlmuth P, Arnold D, Behr J, Gloeckner C, et al. Clinical outcome, risk assessment, and seasonal variation in hospitalized COVID-19 patients-Results from the CORONA Germany study. PloS one. 2021;16(6):e0252867. doi: 10.1371/journal.pone.0252867 34138888 PMC8211271

[30] Fan G, Yang Z, Lin Q, Zhao S, Yang L, He D. Decreased Case Fatality Rate of COVID-19 in the Second Wave: A study in 53 countries or regions. Transboundary and emerging diseases. 2021;68(2):213–5. doi: 10.1111/tbed.13819 32892500

[31] Sweden PHAo. Vaccination coverage and reported cases (Vaccinationstäckning och rapporterade sjukdomsfall). 2021 2021-05-20.

[32] Vekaria B, Overton C, Wisniowski A, Ahmad S, Aparicio-Castro A, Curran-Sebastian J, et al. Hospital length of stay for COVID-19 patients: Data-driven methods for forward planning. BMC Infect Dis. 2021;21(1):700. doi: 10.1186/s12879-021-06371-6 34294037 PMC8295642

[33] Hägg S, Jylhävä J, Wang Y, Xu H, Metzner C, Annetorp M, et al. Age, Frailty, and Comorbidity as Prognostic Factors for Short-Term Outcomes in Patients With Coronavirus Disease 2019 in Geriatric Care. J Am Med Dir Assoc. 2020;21(11):1555–9.e2. doi: 10.1016/j.jamda.2020.08.014 32978065 PMC7427570

[34] "Swedish Society of Infectious disease specialists SHsaSfCM. National care program for suspected or confirmed COVID-19 (Nationellt vårdprogram för misstänkt och bekräftad covid-19). 2021.

[35] Commission C. Sweden during the pandemic. ISBN 978-91-525-0333-1

[36] commission C. Sweden during the pandemic. In: Investigations OG, editor.

[37] IBRD+IDA" TWB. Hospital beds (per 1000 people)- Sweden 2022 [Data from the World Health Organization, supplemented by country data. 1960–2018]. Available from: https://data.worldbank.org/indicator/SH.MED.BEDS.ZS?end=2018&locations=SE&start=1960&view=chart.

