# Supplementary material for: Temporal trends in hospitalizations and 30-day mortality in older patients during the COVID pandemic from March 2020 to July 2021
Source: PLoS One. 2023 Sep 14;18(9):e0291237. doi: 10.1371/journal.pone.0291237 (PMC10501674; doi:10.1371/journal.pone.0291237)
Supplement: S1 Table — (DOCX) [file pone.0291237.s001.docx]

**S1 Table. Hospitalizations for COVID-19 and other causes in patients 70 years old and over in geriatric clinics**

|  | **2020-2021** | **2020** | **2020** | **2020** | **2020** | **2020** | **2021** | **2021** | **2021** |
| --- | --- | --- | --- | --- | --- | --- | --- | --- | --- |
|  | **Overall** | **Mar-Apr** | **May-June** | **July-Aug** | **Sep-Oct** | **Nov-Dec** | **Jan-Feb** | **Mar-Apr** | **May-June-July** |
| **COVID-19** |  |  |  |  |  |  |  |  |  |
| **N** | 5320 | 990 | 813 | 117 | 118 | 1329 | 838 | 870 | 245 |
| **Age, year** | 84.0 (78.0, 89.0) | 84.5 (79.0, 90.0) | 85.0 (79.0, 91.0) | 86.0 (81.0, 90.0) | 85.5 (81.0, 91.0) | 84.0 (79.0, 89.0) | 83.0 (78.0, 89.0) | 81.0 (76.0, 86.0) | 81.0 (75.0, 88.0) |
| **Women** | 2822 (53.0%) | 535 (54.0%) | 442 (54.4%) | 63 (53.8%) | 70 (59.3%) | 723 (54.4%) | 429 (51.2%) | 436 (50.1%) | 124 (50.6%) |
| **CCI** | 2.0 (0.0, 3.0) | 2.0 (0.0, 3.0) | 2.0 (0.0, 3.0) | 1.0 (0.0, 3.0) | 1.0 (0.0, 3.0) | 2.0 (0.0, 3.0) | 2.0 (0.0, 3.0) | 2.0 (0.0, 3.0) | 2.0 (0.0, 3.0) |
| **Hypertension** | 1777 (33.4%) | 380 (38.4%) | 310 (38.1%) | 25 (21.4%) | 43 (36.4%) | 383 (28.8%) | 268 (32.0%) | 306 (35.2%) | 62 (25.3%) |
| **Diabetes** | 2014 (37.9%) | 344 (34.7%) | 287 (35.3%) | 33 (28.2%) | 32 (27.1%) | 510 (38.4%) | 331 (39.5%) | 367 (42.2%) | 110 (44.9%) |
| **Chronicheart failure** | 761 (14.3%) | 179 (18.1%) | 152 (18.7%) | 11 (9.4%) | 21 (17.8%) | 176 (13.2%) | 115 (13.7%) | 85 (9.8%) | 22 (9.0%) |
| **Myocardial Infarction** | 233 (4.4%) | 43 (4.3%) | 31 (3.8%) | 1 (0.9%) | 4 (3.4%) | 57 (4.3%) | 39 (4.7%) | 44 (5.1%) | 14 (5.7%) |
| **Chronical pulmonary disease** | 709 (13.3%) | 148 (14.9%) | 108 (13.3%) | 9 (7.7%) | 20 (16.9%) | 185 (13.9%) | 114 (13.6%) | 106 (12.2%) | 19 (7.8%) |
| **Asthma** | 185 (3.5%) | 36 (3.6%) | 24 (3.0%) | 3 (2.6%) | 8 (6.8%) | 41 (3.1%) | 28 (3.3%) | 39 (4.5%) | 6 (2.4%) |
| **Cancer** | 331 (6.2%) | 74 (7.5%) | 49 (6.0%) | 16 (13.7%) | 10 (8.5%) | 84 (6.3%) | 41 (4.9%) | 42 (4.8%) | 15 (6.1%) |
| **Stroke** | 307 (5.8%) | 66 (6.7%) | 58 (7.1%) | 4 (3.4%) | 9 (7.6%) | 79 (5.9%) | 38 (4.5%) | 39 (4.5%) | 14 (5.7%) |
| **Atrial fibrillation** | 1061 (19.9%) | 217 (21.9%) | 195 (24.0%) | 25 (21.4%) | 26 (22.0%) | 273 (20.5%) | 151 (18.0%) | 139 (16.0%) | 35 (14.3%) |
| **Initial S02<90%** | 342 (6.4%) | 89 (9.0%) | 39 (4.8%) | 3 (2.6%) | 7 (5.9%) | 99 (7.4%) | 50 (6.0%) | 42 (4.8%) | 13 (5.3%) |
| **ACEI** | 1229 (23.1%) | 246 (24.8%) | 198 (24.4%) | 21 (17.9%) | 24 (20.3%) | 276 (20.8%) | 204 (24.3%) | 190 (21.8%) | 70 (28.6%) |
| **ARB** | 1462 (27.5%) | 251 (25.4%) | 209 (25.7%) | 32 (27.4%) | 40 (33.9%) | 378 (28.4%) | 243 (29.0%) | 248 (28.5%) | 61 (24.9%) |
| **β-blocker** | 2745 (51.6%) | 531 (53.6%) | 419 (51.5%) | 65 (55.6%) | 54 (45.8%) | 681 (51.2%) | 436 (52.0%) | 430 (49.4%) | 129 (52.7%) |
| **CCB** | 1621 (30.5%) | 295 (29.8%) | 237 (29.2%) | 34 (29.1%) | 24 (20.3%) | 412 (31.0%) | 255 (30.4%) | 286 (32.9%) | 78 (31.8%) |
| **Diuretics** | 2819 (53.0%) | 563 (56.9%) | 449 (55.2%) | 66 (56.4%) | 74 (62.7%) | 672 (50.6%) | 453 (54.1%) | 410 (47.1%) | 132 (53.9%) |
| **Statins** | 2148 (40.4%) | 380 (38.4%) | 309 (38.0%) | 40 (34.2%) | 48 (40.7%) | 548 (41.2%) | 354 (42.2%) | 380 (43.7%) | 89 (36.3%) |
| **Warfarin** | 388 (7.3%) | 85 (8.6%) | 61 (7.5%) | 15 (12.8%) | 7 (5.9%) | 93 (7.0%) | 62 (7.4%) | 48 (5.5%) | 17 (6.9%) |
| **LMWH** | 2732 (51.4%) | 410 (41.4%) | 427 (52.5%) | 39 (33.3%) | 59 (50.0%) | 772 (58.1%) | 417 (49.8%) | 509 (58.5%) | 99 (40.4%) |
| **NOAC** | 1745 (32.8%) | 262 (26.5%) | 285 (35.1%) | 41 (35.0%) | 34 (28.8%) | 429 (32.3%) | 315 (37.6%) | 287 (33.0%) | 92 (37.6%) |
| **Glucocorticoids** | 1492 (28.0%) | 151 (15.3%) | 148 (18.2%) | 23 (19.7%) | 31 (26.3%) | 465 (35.0%) | 250 (29.8%) | 338 (38.9%) | 86 (35.1%) |
| **Antibiotics** | 1798 (33.8%) | 369 (37.3%) | 252 (31.0%) | 41 (35.0%) | 41 (34.7%) | 426 (32.1%) | 280 (33.4%) | 288 (33.1%) | 101 (41.2%) |
| **Days of hospitalization** | 9.0 (6.0, 13.0) | 9.0 (6.0, 14.0) | 9.0 (6.0, 14.0) | 9.0 (6.0, 13.0) | 11.0 (8.0, 19.0) | 9.0 (6.0, 14.0) | 8.0 (6.0, 12.0) | 7.0 (5.0, 11.0) | 8.0 (5.0, 10.0) |
| **30-day mortality** | 910 (17.1%) | 282 (28.5%) | 134 (16.5%) | 8 (6.8%) | 21 (17.8%) | 228 (17.2%) | 106 (12.6%) | 99 (11.4%) | 32 (13.1%) |
| **Non-COVID** |  |  |  |  |  |  |  |  |  |
| **N** | 32243 | 3177 | 3198 | 4191 | 4736 | 2770 | 3217 | 4028 | 6926 |
| **Age, year** | 85.0 (79.0, 90.0) | 86.0 (80.0, 91.0) | 86.0 (80.0, 91.0) | 85.0 (79.0, 90.0) | 85.0 (79.0, 90.0) | 85.0 (80.0, 90.0) | 85.0 (79.0, 90.0) | 85.0 (79.0, 90.0) | 85.0 (79.0, 90.0) |
| **Women** | 19336 (60.0%) | 1895 (59.6%) | 1900 (59.4%) | 2574 (61.4%) | 2822 (59.6%) | 1635 (59.0%) | 1907 (59.3%) | 2429 (60.3%) | 4174 (60.3%) |
| **CCI** | 1.0 (0.0, 3.0) | 2.0 (0.0, 3.0) | 1.0 (0.0, 3.0) | 1.0 (0.0, 3.0) | 1.0 (0.0, 3.0) | 1.0 (0.0, 3.0) | 1.0 (0.0, 3.0) | 1.0 (0.0, 3.0) | 1.0 (0.0, 3.0) |
| **Hypertension** | 9804 (30.4%) | 1150 (36.2%) | 1050 (32.8%) | 1305 (31.1%) | 1388 (29.3%) | 817 (29.5%) | 930 (28.9%) | 1138 (28.3%) | 2026 (29.3%) |
| **Diabetes** | 9080 (28.2%) | 908 (28.6%) | 844 (26.4%) | 1174 (28.0%) | 1360 (28.7%) | 769 (27.8%) | 887 (27.6%) | 1147 (28.5%) | 1991 (28.7%) |
| **Chronic heart failure** | 5001 (15.5%) | 668 (21.0%) | 541 (16.9%) | 627 (15.0%) | 680 (14.4%) | 430 (15.5%) | 480 (14.9%) | 622 (15.4%) | 953 (13.8%) |
| **Myocardial Infarction** | 1206 (3.7%) | 160 (5.0%) | 123 (3.8%) | 163 (3.9%) | 162 (3.4%) | 102 (3.7%) | 103 (3.2%) | 140 (3.5%) | 253 (3.7%) |
| **Chronical pulmonary disease** | 3189 (9.9%) | 447 (14.1%) | 379 (11.9%) | 417 (9.9%) | 446 (9.4%) | 225 (8.1%) | 313 (9.7%) | 345 (8.6%) | 617 (8.9%) |
| **Asthma** | 538 (1.7%) | 76 (2.4%) | 60 (1.9%) | 56 (1.3%) | 74 (1.6%) | 46 (1.7%) | 48 (1.5%) | 70 (1.7%) | 108 (1.6%) |
| **Cancer** | 2173 (6.7%) | 266 (8.4%) | 246 (7.7%) | 275 (6.6%) | 289 (6.1%) | 186 (6.7%) | 219 (6.8%) | 266 (6.6%) | 426 (6.2%) |
| **Stroke** | 2254 (7.0%) | 257 (8.1%) | 289 (9.0%) | 316 (7.5%) | 327 (6.9%) | 188 (6.8%) | 224 (7.0%) | 282 (7.0%) | 371 (5.4%) |
| **Atrial fibrillation** | 6578 (20.4%) | 896 (28.2%) | 765 (23.9%) | 851 (20.3%) | 942 (19.9%) | 557 (20.1%) | 638 (19.8%) | 714 (17.7%) | 1215 (17.5%) |
| **Initial S02<90%** | 991 (3.1%) | 135 (4.2%) | 100 (3.1%) | 116 (2.8%) | 153 (3.2%) | 90 (3.2%) | 87 (2.7%) | 134 (3.3%) | 176 (2.5%) |
| **ACEI** | 7445 (23.1%) | 745 (23.4%) | 772 (24.1%) | 924 (22.0%) | 1049 (22.1%) | 673 (24.3%) | 768 (23.9%) | 940 (23.3%) | 1574 (22.7%) |
| **ARB** | 8602 (26.7%) | 803 (25.3%) | 836 (26.1%) | 1120 (26.7%) | 1274 (26.9%) | 737 (26.6%) | 805 (25.0%) | 1084 (26.9%) | 1943 (28.1%) |
| **β -blocker** | 17298 (53.6%) | 1744 (54.9%) | 1720 (53.8%) | 2231 (53.2%) | 2515 (53.1%) | 1445 (52.2%) | 1691 (52.6%) | 2202 (54.7%) | 3750 (54.1%) |
| **CCB** | 9828 (30.5%) | 947 (29.8%) | 955 (29.9%) | 1240 (29.6%) | 1404 (29.6%) | 847 (30.6%) | 1018 (31.6%) | 1245 (30.9%) | 2172 (31.4%) |
| **Diuretics** | 17667 (54.8%) | 1740 (54.8%) | 1746 (54.6%) | 2229 (53.2%) | 2562 (54.1%) | 1475 (53.2%) | 1745 (54.2%) | 2275 (56.5%) | 3895 (56.2%) |
| **Statins** | 12580 (39.0%) | 1181 (37.2%) | 1272 (39.8%) | 1541 (36.8%) | 1810 (38.2%) | 1077 (38.9%) | 1328 (41.3%) | 1569 (39.0%) | 2802 (40.5%) |
| **Warfarin** | 2440 (7.6%) | 282 (8.9%) | 252 (7.9%) | 302 (7.2%) | 361 (7.6%) | 188 (6.8%) | 283 (8.8%) | 312 (7.7%) | 460 (6.6%) |
| **LMWH** | 5960 (18.5%) | 622 (19.6%) | 598 (18.7%) | 786 (18.8%) | 798 (16.8%) | 584 (21.1%) | 643 (20.0%) | 783 (19.4%) | 1146 (16.5%) |
| **NOAC** | 9975 (30.9%) | 942 (29.7%) | 1012 (31.6%) | 1245 (29.7%) | 1463 (30.9%) | 846 (30.5%) | 959 (29.8%) | 1245 (30.9%) | 2263 (32.7%) |
| **Glucocorticoids** | 5426 (16.8%) | 585 (18.4%) | 515 (16.1%) | 675 (16.1%) | 802 (16.9%) | 439 (15.8%) | 521 (16.2%) | 669 (16.6%) | 1220 (17.6%) |
| **Antibiotics** | 11154 (34.6%) | 1071 (33.7%) | 1000 (31.3%) | 1360 (32.5%) | 1547 (32.7%) | 986 (35.6%) | 1111 (34.5%) | 1469 (36.5%) | 2610 (37.7%) |
| **Days of hospitalization** | 6.0 (4.0, 9.0) | 6.0 (4.0, 8.0) | 7.0 (4.0, 9.0) | 7.0 (4.0, 9.0) | 6.0 (4.0, 9.0) | 7.0 (5.0, 9.0) | 7.0 (5.0, 9.0) | 7.0 (4.0, 9.0) | 6.0 (4.0, 8.0) |
| **30-day mortality** | 2022 (6.3%) | 309 (9.7%) | 199 (6.2%) | 263 (6.3%) | 225 (4.8%) | 193 (7.0%) | 209 (6.5%) | 231 (5.7%) | 393 (5.7%) |

**Abbreviations: S02<90% peripheral oxygen saturation under 90%; ACEI, angiotensin-converting enzyme inhibitors; ARB, angiotensin receptor blockers; CCB, calcium channel blockers; LMWH, low-molecular-weight heparin; NOAC, Non-vitamin-K antagonist oral anticoagulant. Days of hospitalization: median number of days hospitalized (interquartile range). 30-day mortality is calculated from the date of hospitalization.**
