# Supplementary material for: Temporal trends in hospitalizations and 30-day mortality in older patients during the COVID pandemic from March 2020 to July 2021
Source: PLoS One. 2023 Sep 14;18(9):e0291237. doi: 10.1371/journal.pone.0291237 (PMC10501674; doi:10.1371/journal.pone.0291237)
Supplement: S2 Table — (DOCX) [file pone.0291237.s002.docx]

**S2 Table COVID-19 cases, mortality and hospitalizations in Stockholm and in geriatric clinics for the population 70 years old and above.**

| **Week** | **COVID-19 positive cases in Stockholm** | **30-day COVID-19 mortality Stockholm** | **COVID-19 hospitalizations in Stockholm** | **Total hospitalizations in Stockholm** | **COVID-19 geriatric hospitalizations** | **30-day mortality after geriatric hospitalization for COVID-19** | **Other diagnoses, geriatric hospitalizations** | **30-day mortality after geriatric hospitalization, non-COVID-19** | **Total geriatric hospitalizations** |
| --- | --- | --- | --- | --- | --- | --- | --- | --- | --- |
| March 1, 2020 |  |  |  |  |  |  | 28 | 3 | 28 |
| March 2, 2020 |  |  | 11 | 2 718 | 6 | 0 | 642 | 53 | 648 |
| March 9, 2020 | 40 | X | 67 | 2 515 | 15 | 4 | 529 | 51 | 544 |
| March 16, 2020 | 231 | 22 | 268 | 2 364 | 38 | 17 | 417 | 49 | 455 |
| March 23, 2020 | 557 | 102 | 550 | 2 237 | 111 | 39 | 307 | 40 | 418 |
| March 30, 2020 | 675 | 213 | 724 | 2 130 | 178 | 67 | 264 | 18 | 442 |
| April 6, 2020 | 763 | 345 | 735 | 2 120 | 175 | 46 | 256 | 33 | 431 |
| April 13, 2020 | 709 | 305 | 643 | 2 116 | 172 | 47 | 246 | 28 | 418 |
| April 20, 2020 | 598 | 233 | 620 | 2 260 | 187 | 41 | 280 | 17 | 467 |
| April 27, 2020 | 420 | 207 | 465 | 2 030 | 141 | 25 | 278 | 21 | 419 |
| May 4, 2020 | 392 | 147 | 464 | 2 245 | 144 | 28 | 311 | 28 | 455 |
| May 11, 2020 | 293 | 117 | 412 | 2 120 | 141 | 13 | 324 | 32 | 465 |
| May 18, 2020 | 286 | 85 | 322 | 1 999 | 112 | 26 | 333 | 18 | 445 |
| May 25, 2020 | 262 | 67 | 302 | 2 186 | 97 | 25 | 346 | 24 | 443 |
| June 1, 2020 | 240 | 77 | 289 | 2 277 | 94 | 15 | 409 | 25 | 503 |
| June 8, 2020 | 196 | 49 | 240 | 2 320 | 88 | 14 | 401 | 19 | 489 |
| June 15, 2020 | 154 | 42 | 149 | 2 081 | 51 | 2 | 392 | 19 | 443 |
| June 22, 2020 | 131 | 27 | 124 | 2 235 | 42 | 6 | 445 | 21 | 487 |
| June 29, 2020 | 53 | 18 | 87 | 2 187 | 35 | 3 | 474 | 33 | 509 |
| July 6, 2020 | 40 | 11 | 57 | 2 125 | 28 | 1 | 487 | 28 | 515 |
| July 13, 2020 | 31 | 7 | 44 | 2 018 | 12 | 1 | 469 | 26 | 481 |
| July 20, 2020 | 35 | 7 | 38 | 2 010 | 10 | 1 | 471 | 28 | 481 |
| July 27, 2020 | 18 | 4 | 37 | 2 078 | 12 | 0 | 464 | 50 | 476 |
| August 3, 2020 | 27 | 5 | 29 | 2 136 | 12 | 1 | 468 | 26 | 480 |
| August 10, 2020 | 19 | X | 21 | 2 145 | 9 | x | 476 | 31 | 485 |
| August 17, 2020 | 12 | 5 | 17 | 2 336 | 6 | 0 | 494 | 29 | 500 |
| August 24, 2020 | 9 | X | 11 | 2 453 | 4 | 0 | 494 | 19 | 498 |
| August 31, 2020 | 15 | X | 19 | 2 420 | 3 | 0 | 455 | 23 | 458 |
| September 7, 2020 | 6 | X | 10 | 2 552 | 2 | x | 535 | 20 | 537 |
| September 14, 2020 | 33 | X | 16 | 2 567 | 1 | 0 | 542 | 35 | 543 |
| September 21, 2020 | 55 | X | 24 | 2 552 | 4 | x | 542 | 22 | 546 |
| September 28, 2020 | 64 | X | 25 | 2 673 | 6 | 0 | 567 | 21 | 573 |
| October 5, 2020 | 74 | 10 | 59 | 2 726 | 11 | 1 | 565 | 28 | 576 |
| October 12, 2020 | 93 | X | 73 | 2 655 | 18 | 3 | 581 | 32 | 599 |
| October 19, 2020 | 141 | 10 | 87 | 2 592 | 17 | 5 | 540 | 28 | 557 |
| October 26, 2020 | 295 | 17 | 196 | 2 683 | 60 | 10 | 492 | 19 | 552 |
| November 2, 2020 | 626 | 32 | 287 | 2 545 | 71 | 19 | 432 | 33 | 503 |
| November 9, 2020 | 755 | 66 | 404 | 2 433 | 108 | 22 | 351 | 22 | 459 |
| November 16, 2020 | 864 | 85 | 542 | 2 565 | 137 | 22 | 318 | 19 | 455 |
| November 23, 2020 | 842 | 92 | 560 | 2 579 | 173 | 31 | 327 | 18 | 500 |
| November 30, 2020 | 818 | 104 | 552 | 2 596 | 174 | 27 | 289 | 25 | 463 |
| December 7, 2020 | 830 | 108 | 583 | 2 680 | 183 | 29 | 301 | 19 | 484 |
| December 14, 2020 | 857 | 124 | 608 | 2 590 | 175 | 34 | 302 | 20 | 477 |
| December 21, 2020 | 634 | 104 | 471 | 2 247 | 172 | 26 | 252 | 24 | 424 |
| December 28, 2020 | 653 | 91 | 433 | 2 308 | 156 | 22 | 229 | 17 | 385 |
| January 4, 2021 | 540 | 83 | 431 | 2 361 | 148 | 22 | 292 | 17 | 440 |
| January 11, 2021 | 410 | 70 | 338 | 2 501 | 142 | 23 | 348 | 22 | 490 |
| January 18, 2021 | 357 | 68 | 278 | 2 512 | 116 | 16 | 370 | 28 | 486 |
| January 25, 2021 | 252 | 56 | 218 | 2 475 | 107 | 14 | 412 | 29 | 519 |
| February 1, 2021 | 225 | 41 | 173 | 2 403 | 69 | 5 | 416 | 31 | 485 |
| February 8, 2021 | 220 | 33 | 117 | 2 363 | 68 | 7 | 425 | 19 | 493 |
| February 15, 2021 | 261 | 21 | 176 | 2 513 | 91 | 7 | 441 | 27 | 532 |
| February 22, 2021 | 298 | 17 | 179 | 2 504 | 73 | 8 | 460 | 31 | 533 |
| March 1, 2021 | 339 | 30 | 188 | 2 446 | 83 | 10 | 463 | 32 | 546 |
| March 8, 2021 | 367 | 23 | 233 | 2 576 | 87 | 12 | 481 | 33 | 568 |
| March 15, 2021 | 446 | 30 | 256 | 2 510 | 100 | 12 | 464 | 16 | 564 |
| March 22, 2021 | 459 | 30 | 284 | 2 664 | 118 | 13 | 462 | 26 | 580 |
| March 29, 2021 | 459 | 33 | 259 | 2 337 | 105 | 11 | 468 | 19 | 573 |
| April 5, 2021 | 434 | 21 | 259 | 2 357 | 101 | 12 | 394 | 28 | 495 |
| April 12, 2021 | 419 | 31 | 267 | 2 435 | 120 | 12 | 443 | 30 | 563 |
| April 19, 2021 | 347 | 30 | 201 | 2 376 | 92 | 7 | 459 | 21 | 551 |
| April 26, 2021 | 246 | 23 | 147 | 2 463 | 74 | 12 | 482 | 28 | 556 |
| May 3, 2021 | 211 | 19 | 136 | 2 529 | 46 | 4 | 468 | 30 | 514 |
| May 10, 2021 | 142 | 15 | 77 | 2 318 | 39 | 6 | 489 | 27 | 528 |
| May 17, 2021 | 84 | 13 | 71 | 2 582 | 30 | 3 | 518 | 28 | 548 |
| May 24, 2021 | 59 | 8 | 40 | 2 469 | 25 | 3 | 510 | 35 | 535 |
| May 31, 2021 | 12 | 5 | 26 | 2 537 | 17 | 3 | 529 | 26 | 546 |
| June 7, 2021 | 17 | X | 16 | 2 462 | 8 | 0 | 515 | 16 | 523 |
| June 14, 2021 | 19 | X | 12 | 2 553 | 14 | 1 | 567 | 30 | 581 |
| June 21, 2021 | 5 | X | 12 | 2 289 | 17 | 2 | 513 | 22 | 530 |
| June 28, 2021 | 11 | X | 6 | 2 362 | 7 | x | 544 | 30 | 551 |
| July 5, 2021 | 6 | 0 | 7 | 2 271 | 7 | x | 576 | 39 | 583 |
| July 12, 2021 | 8 | 0 | 10 | 2 107 | 5 | x | 566 | 43 | 571 |
| July 19, 2021 | 16 | 0 | 7 | 2 105 | 9 | x | 526 | 32 | 535 |
| July 26, 2021 | 23 | 0 | 12 | 2 134 | 11 | x | 517 | 33 | 528 |
